# Full Chloroplast Genome Assembly of 11 Diverse Watermelon Accessions

**DOI:** 10.3389/fgene.2017.00046

**Published:** 2017-04-18

**Authors:** Chao Shi, Shuo Wang, Fei Zhao, Hua Peng, Chun-Lei Xiang

**Affiliations:** ^1^Key Laboratory for Plant Diversity and Biogeography of East Asia, Kunming Institute of Botany, Chinese Academy of SciencesKunming, China; ^2^Faculty of Life Science and Technology, Kunming University of Science and TechnologyKunming, China

**Keywords:** chloroplast genome, watermelon, genome assembly, annotation, cucurbitaceae

## Introduction

Watermelon [*Citrullus lanatus* (Thunb.) Matsum and Nakai] is an important cucurbit crop of the family Cucurbitaceae. The large edible watermelon fruits contribute to the diet of consumers throughout the world and the great number of consumption (about 90 million tons every year) makes it among the top five most consumed fresh fruits (http://www.fao.org/faostat/en/#home). It supplies people with not only large amount of water but also important nutritional compounds, such as sugars, lycopene, and cardiovascular health promoting amino acids (Hayashi et al., 2005; Collins et al., 2007). The domestication of wild *C. lanatus* and its worldwide cultivation have resulted in many modern watermelon varieties with diverse fruit shapes, sizes, color, texture, flavor, and nutrient compositions (Erickson et al., 2005).

Human domestication and the breeding of crops from wild to cultivated groups have long been an important issue in plant science (Meyer and Purugganan, 2013). *C. lanatus* can serve as a good model species for studying this process, since it includes three subspecies corresponding to wild, semi-wild, and cultivated groups (Fursa, 1972): the wild subspecies *C. lanatus* subsp. *lanatus*, which represents for an ancient subspecies group that has natural populations in southern Africa; the semi-wild subspecies *C. lanatus* subsp. *mucosospermus* Fursa, which represents the egusi watermelon group that contains large seeds in the edible fleshy pericarp; and the cultivated subspecies *C. lanatus* subsp. *vulgaris* Fursa, which represents the sweet (dessert) watermelon groups (including East-Asia ecotype and America ecotype) that give rise to the modern cultivated watermelon (Erickson et al., 2005). Previous study has revealed important genome-wide changes under human domestication and breeding (Guo et al., 2013), while the sequence variations of chloroplast genome underwent this process has not been reported.

Chloroplast genomes contribute a lot to plant genetic diversity and evolutionary studies (Green, 2011). The chloroplast genomes contain both conserved and variable protein-coding genes that can resolve phylogenetic relationships at either high (Jansen et al., 2007; Moore et al., 2007, 2010) or low taxonomic levels (Parks et al., 2009; Carbonell-Caballero et al., 2015). They also include highly variable non-genic markers that are widely used in plant barcoding (Taberlet et al., 2007; Dong et al., 2012) and population studies (Doorduin et al., 2011). In this study, we report the complete chloroplast genome sequences of 11 watermelon accessions representing morphologically and genetically differentiated taxa of all the three subspecies. As a continuation and supplementary of the watermelon nuclear genome sequencing project (Guo et al., 2013), these chloroplast genome sequences will further expand the genome resources for watermelon genetic studies.

## Materials and methods

All 11 watermelon accessions in this study were from the watermelon nuclear genome sequencing project and all plant materials were conserved in Beijing Academy of Agriculture and Forestry Sciences, Beijing, China (Guo et al., 2013). The DNA was extracted from fresh leaves of these materials and the Illumina sequencing libraries construction, sequencing was prepared following sequencer's instructions as previously described (Guo et al., 2013). The sequenced Illumina paired-end sequence reads (2 × 100 bp in length; FASTQ format) were ranged from 1.1 to 2.1 GB. The 11 representative watermelon accessions included five major cultivated varieties of *C. lanatus* subsp. *vulgaris* (two East-Asia and three America ecotypes), three semi-wild varieties of *C. lanatus* subsp. *mucosospermus*, and three wild varieties of *C. lanatus* subsp. *lanatus* (Table 1).

**Table 1:** **Chloroplast genome informations for 11 watermelon accessions in this study**.

**Accessions**	**Species**	**Groups**	**Chloroplast genome size**
JX-2	*C. lanatus* subsp. *vulgaris* East-Asia ecotyp	Cultivated watermelon	156,907
JLM			156,907
Black diamo	*C. lanatus* subsp. *vulgaris* America ecotype		156,906
Calhoun gray			156,907
Sugarlee			156,906
PI1893	*C. lanatus subsp. mucosospermus*	Semi-wild watermleon	156,905
PI500301			156,905
PI249010			156,907
PI4822	*C. lanatus* subsp. *lanatus*	Wild watermelon	156,699
PI482303			156,886
PI482326			156,891

Before assembly, the obtained Illumina paired-end total DNA sequencing data of each accession were subjected to NCBI-blast version 2.2.31+ (ftp://ftp.ncbi.nih.gov/blast/) to screen out chloroplast DNA reads with a reference data set contained all the sequenced angiosperm chloroplast genome sequences so far (ftp://ftp.ncbi.nlm.nih.gov/refseq/release/plastid/). The filtered chloroplast DNA data were then subjected to SOAPdenovo2 (Luo et al., 2012), ABySS version 1.9.0 (Simpson et al., 2009), and SPAdes version 3.1.0 (Bankevich et al., 2012) for several runs of *de novo* assembly until it resulted in one final circular contig (FASTA format) for each accession. Annotation was performed with DualOrganellarGenomeAnnotator (DOGMA) (Wyman et al., 2004) using default parameters to predict protein-coding genes, tRNA genes, and ribosomal RNA (rRNA) genes. For genes with low sequence identity, manual annotation was performed to determine the positions of start and stop codons depending on the translated amino acid sequence using the chloroplast/bacterial genetic code. The final GenBank format annotation information was produced using Sequin (http://www.ncbi.nlm.nih.gov/). All these records with Fasta and GenBank formats were then deposited and can be viewed in National Center for Biotechnology Information (NCBI) database (http://www.ncbi.nlm.nih.gov/nuccore).

## Results and discussion

**Figure 1:**
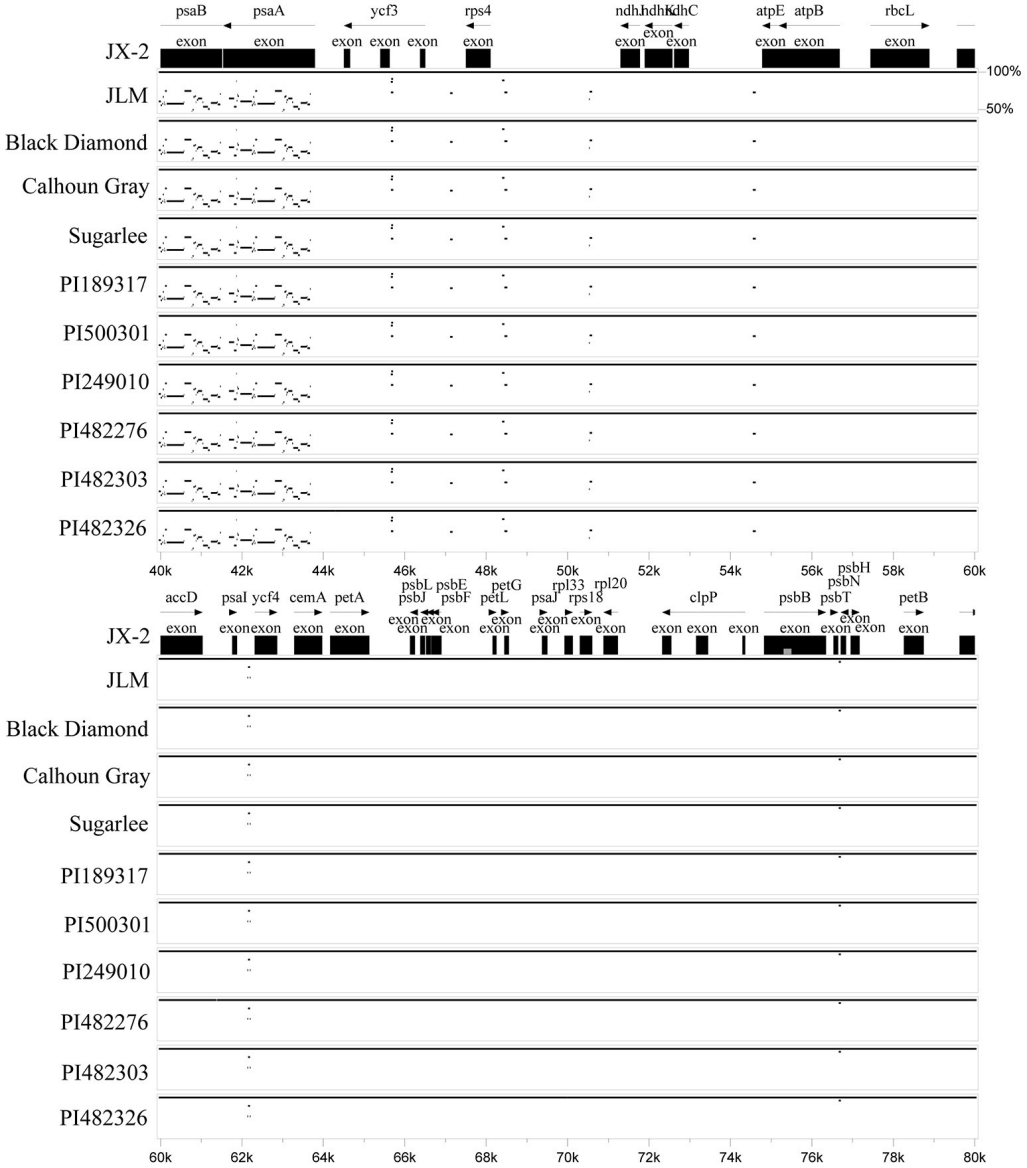
**Visualization of sequence alignments among the 11 watermelon chloroplast genomes**. VISTA-based identity plots show sequence identity among the 11 sequenced chloroplast genomes with JX-2 as a reference. The genomic coding regions ranging from 40 to 80 Kbp were indicated as black boxes.

Sizes of the 11 determined chloroplast genomes of each watermelon accession varied from 156,699 bp of PI482276 to 156,907 bp of JX-2, JLM, Calhoun Gray, and PI249010 (Table 1). All the chloroplast genomes exhibited a typical quadripartite structure, consisting of a pair of inverted repeat regions (IRs) (25,989–26,108 bp) separated by a large single copy region (LSC) (86,472–86,633 bp) and a small single copy region (SSC) (18,187–18,289 bp). These chloroplast genomes encoded an identical set of 133 genes with 19 of which were duplicated in the IR regions and 114 are unique. Among these unique genes, 15 included one intron and two contain two introns. All of these coding regions account for 51.2–51.7% of the whole genome. Sequence similarities among these species were high (average 99.5%), whereas moderate genome sequence variations were also observed in some genic regions (Figure 1). Three genes, *psaB, psaA*, and *psbA*, which belonged to photosystem I (*psa*) and photosystem II (*psb*) respectively, showed the most sequence variations among all protein-coding genes. In addition, the wild subspecies of *C. lanatus* subsp. *lanatus* group exhibited relatively higher sequence variations than both semi-wild and cultivated groups, which may support the conclusion that human domestication and breeding that target for high yield and desirable fruit qualities have narrowed the genetic diversity of cultivated watermelon (Levi et al., 2001). In all, the chloroplast genome sequences reported in this study will further provide new insights into chloroplast genome variations under human domestication and breeding.

## Deposited data and information to the user

The assembled complete chloroplast genome sequences with annotation information were submitted to NCBI Genbank under the accession numbers KY430683-KY430693 (http://www.ncbi.nlm.nih.gov/nuccore). The raw reads in compressed FASTQ format were deposited at SRA database of NCBI under the accession number SRA052158 (http://www.ncbi.nlm.nih.gov/sra). Users can download and reuse the data for research purpose only with an acknowledgment to us and quoting this paper as reference to the data.

## Author contributions

CS and CX conceived the study and acquired the funding; CS, SW, and FZ performed the genome assembly and analysis; CS, SW, HP, and CX drafted the manuscript. All authors approved the final manuscript.

## Funding

The project was funded by the Youth Innovation Promotion Associaiton, Chinese Academy of Sciences (No. 2013253).

### Conflict of interest statement

The authors declare that the research was conducted in the absence of any commercial or financial relationships that could be construed as a potential conflict of interest.
